# Robotically assisted total gastrectomy for lymphadenopathy after long-term follow-up for multiple type 1 gastric neuroendocrine tumor (NET): a case report

**DOI:** 10.1186/s40792-023-01725-5

**Published:** 2023-08-09

**Authors:** Nozomi Funatsu, Kentaro Hara, Maki Takagi, Atsushi Onodera, Kohdai Ueno, Kazuya Endo, Haruhiko Cho

**Affiliations:** Department of Gastric Surgery, Tokyo Metropolitan Cancer and Infectious Diseases Center, Komagome Hospital, 3-18-22 Honkomagome, Bunkyo-Ku, Tokyo, 113-8677 Japan

**Keywords:** Gastric neuroendocrine tumor, Robotically assisted total gastrectomy, Type A gastritis, Hypergastrinemia

## Abstract

**Background:**

Type 1 gastric neuroendrine tumor (NET) is usually associated with chronic atrophic gastritis and forms multiple lesions. While most cases of type 1 gastric NET are generally slowly growing, some develop regional lymph node metastases even after long-term dormancy.

**Case presentation:**

A 73-year-old male patient with a 32-year history of multiple gastric NET was being followed-up at the study center after endoscopic submucosal dissection (ESD) of a large gastric NET. A blood examination revealed high serum gastrin (> 3000 pg/ml). An endoscopic examination found atrophic mucosa and multiple, elevated lesions in the upper to lower stomach body. Computed tomography (CT) revealed regional lymphadenopathy in the greater omentum along the gastroepiploic artery. Robotically assisted total gastrectomy was performed with D2 lymphadenectomy and Roux-en-Y reconstruction. Pathological analysis revealed a large number of gastric NET (grade 1) with a maximum size of 4.5 mm invading the submucosal layer. A single lymph node metastasis was also detected pathologically at station #4d. The postoperative course was uneventful, and serum gastrin normalized postoperatively. At postoperative year 3, the patient has been doing well without any recurrences.

**Conclusions:**

The present case of multiple gastric NET with a single regional lymph node metastasis at year 32 of follow-up was successfully treated with a robotically assisted total gastrectomy.

## Introduction

The Rindi classification divides gastric neuroendrine tumors (NET) into three types based on their biological behavior. Rindi type I is associated with chronic, atrophic gastritis and has a favorable prognosis with a low frequency of lymph node metastasis (2–5%) and distant organ metastasis (< 2%) [1–3]. The Japanese clinical practice guidelines for gastroenteropancreatic neuroendocrine neoplasms allow observation for Rindi type I gastric NET until local or regional progression develops; however, there is as of yet no method of predicting when a tumor may show progression.

We herein report a case of multiple gastric NET which metastasized to a regional lymph node after 32 years of follow-up and was successfully treated using robotically assisted total gastrectomy.

## Case presentation

A 73-year-old male patient with a 32-year history of hypergastrinemia and multiple gastric NET with a comorbidity of type 2 diabetes mellitus was referred to the study center and found gastric tumors which led to a biopsy-based diagnosis of gastric NET. A blood examination revealed high neuron-specific enolase (NSE) (19.9 ng/ml; normal range: < 16.3 ng/ml) and serum gastrin (> 3000 pg/ml; normal: < 200 pg/ml). Endoscopic examination found atrophic mucosa with erosion and multiple, elevated lesions in the upper to the lower stomach. Based on an analysis of biopsy findings, the lesions were diagnosed as gastric NET. As none of the tumors were large, and no deep-tissue invasion or regional lymphadenopathy was present, the patient elected to receive endoscopic follow-up.

**Fig. 1:**
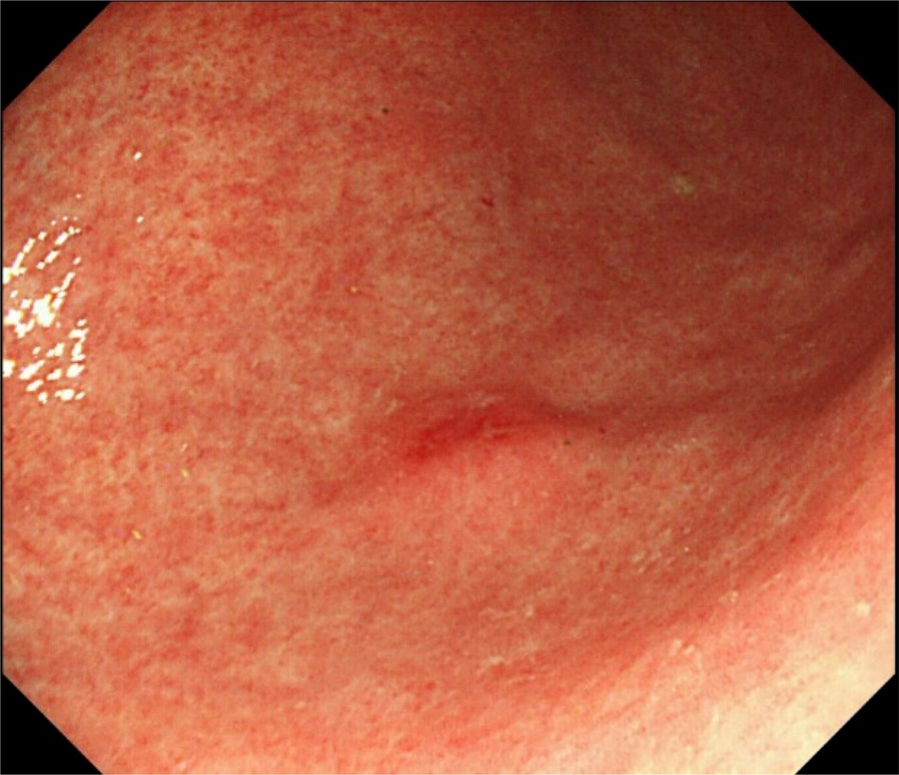
Upper gastrointestinal endoscopic findings. Endoscopy found enlargement in one lesion in the greater curvature of the antrum prompting an endoscopic submucosal dissection

In year 24 of follow-up, he underwent an endoscopic submucosal dissection (ESD) after an endoscopic examination demonstrated enlargement in one lesion in the greater curvature of the antrum (Fig. 1). Histopathological analysis of the dissection specimen led to the diagnosis of pT1(sm) NET with WHO Grade 1 ly0, v0, MIB-1 index 3–4%, and a maximum lesion size of 5 mm. No further treatment was considered necessary at this stage. Endoscopy and computed tomography (CT) were conducted annually to monitor the tumor progression and detect the appearance of any new lesions.

**Fig. 2:**
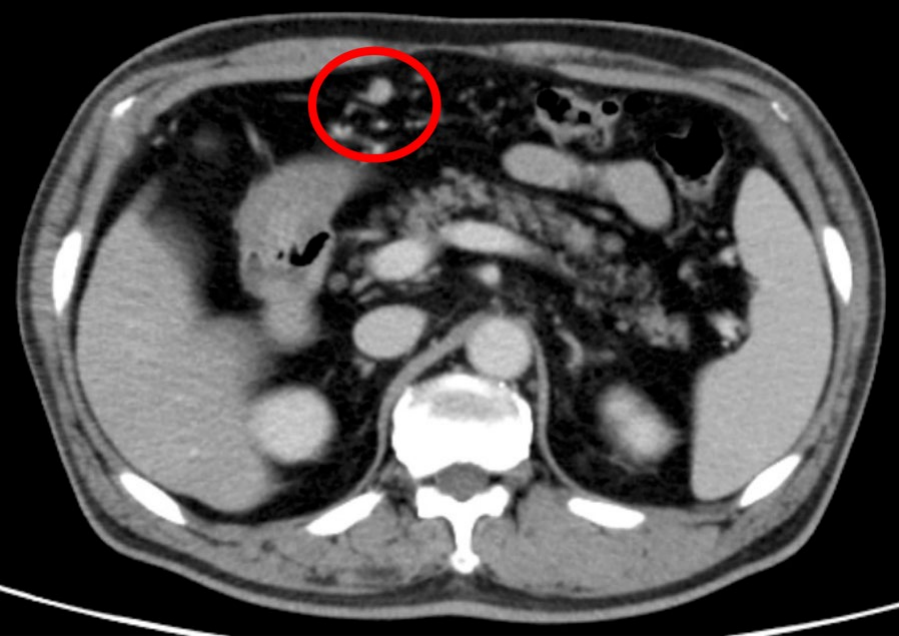
Computed tomography (CT) findings at 32 years of follow-up. CT revealed regional lymphadenopathy with a 10-mm diameter in the greater omentum along the gastroepiploic artery

**Fig. 3:**
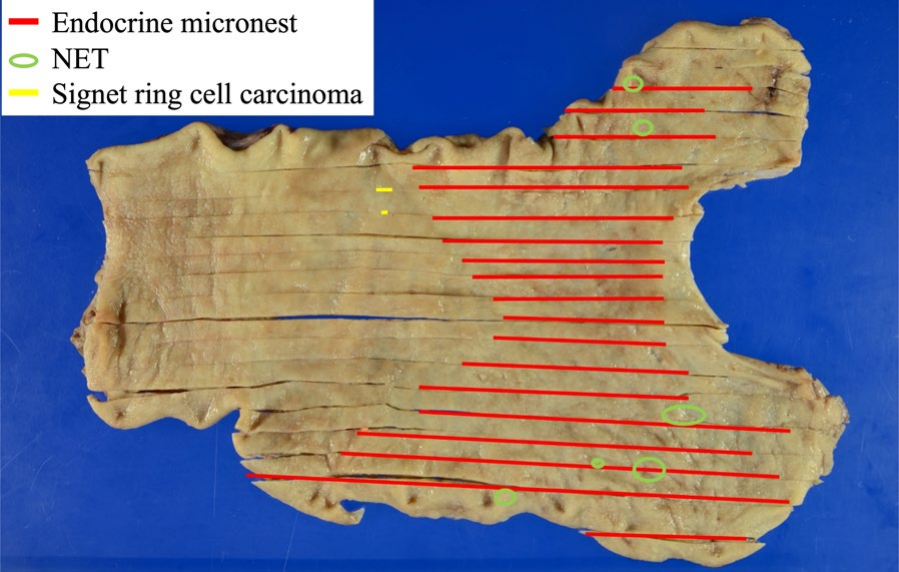
Histopathological findings of a surgical specimen resected in a robotically assisted total gastrectomy. Extensive endocrine micronests were found in the upper to middle gastric body (red lines), and multiple, Grade 1 gastric NET with a maximum size of 4.5 mm were observed (green circles). An early signet ring cell carcinoma was also incidentally found in the middle body of the stomach (yellow lines)

**Fig. 4:**
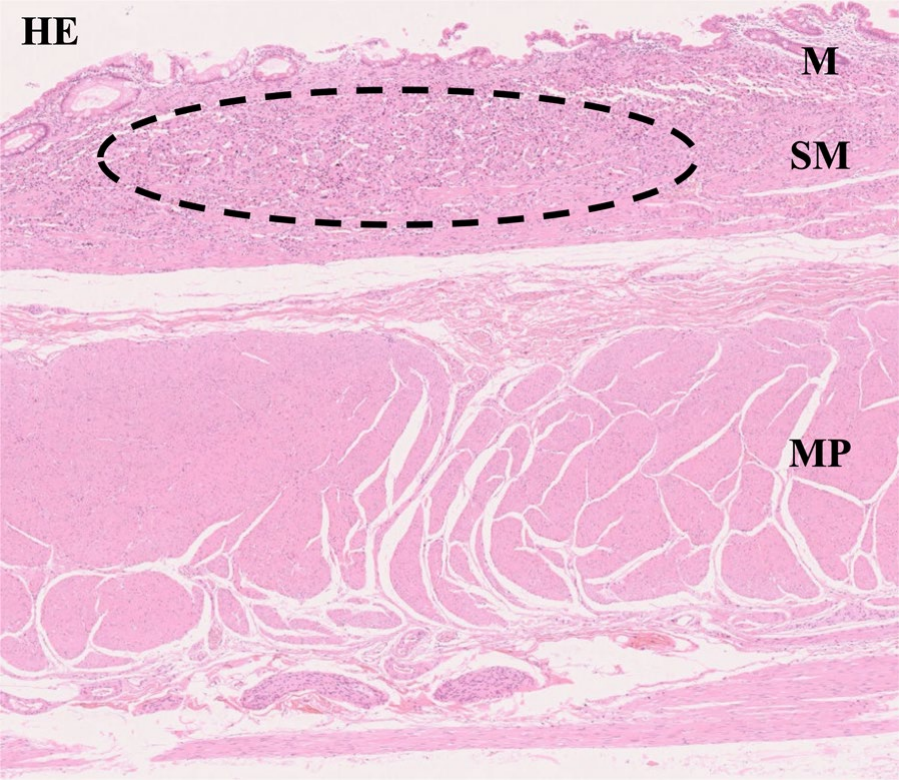
Hematoxylin and eosin staining revealed that the tumor had invaded the submucosal layer

At year 32 of follow-up, CT demonstrated regional lymphadenopathy with a 10-mm diameter in the greater omentum along the gastroepiploic artery (Fig. 2), which was considered to be a lymph node metastasis of the gastric NET. Therefore**,** the patient underwent a robotically assisted total gastrectomy, D2 lymphadenectomy, and Roux-en-Y reconstruction. The operative time was 399 min, and the blood loss was 50 ml. Histopathological examination of a surgical specimen revealed extensive endocrine micronests in the upper to middle gastric body (red lines in Fig. 3) and multiple, Grade 1 gastric NET (green circles in Fig. 3) with ly0, v1a, MIB-1 index 1–2%, and a maximum tumor size of 4.5 mm invading the submucosal layer (Fig. 4). At station #4d (n 1/34), a single lymph node metastasis, whose histological features resembled those of the gastric NET, was detected pathologically. In addition, an early stage signet ring cell carcinoma with ly0, v0 invading the mucosal layer was incidentally found in the middle stomach (yellow lines in Fig. 3). It was not identified during endoscopy but was detected only in the postoperative pathological examination. The patient’s postoperative course was uneventful, and he was discharged on postoperative day 9. Serum gastrin normalized after surgery. The patient has been doing well to date without any recurrences at postoperative year 3.

## Discussion

NET arises from neoplastic changes in neuroendocrine cells and may occur in any organ, although the rectum is the preferred site (55.7% of all sites), followed by the duodenum (16.7%) and the stomach (15.1%). The yearly, age-adjusted incidence of gastric NET is approximately 0.2 per population of 100,000 [1] and has been increasing recently, although NET still remains rare. Gastric NET is classified into three types according to the Rindi classification. Rindi type I is the most common and accounts for approximately 80% of cases. Type II is associated with multiple endocrine neoplasia type-1(MEN-1) with Zollinger–Ellison syndrome and hypergastrinemia, which is a rare cause of gastric NET, having a frequency of 5–6%. Type III is has no specific background and occurs at a frequency of 14–25% [4].

Type I gastric NET is associated with autoimmune atrophic gastritis and hypergastremia. Type A gastritis, corresponding to autoimmune gastritis and thought to be involved in the development of gastric NET, was described by Strickland and Mackey in 1973 [5]. The characteristics of type A gastritis are normal mucosa of the antrum, positive anti-cell wall antibodies, diffuse gastric body inflammation, and severe impairment of gastric acid secretion. Endocrine micronests, which are precursors to NET, arise from gastrin-induced, hyperplastic or neoplastic changes in enterochromaffin-like cells [6]. They frequently occur in the gastric mucosa together with type A gastritis. As the present patient had multiple gastric NET with type A gastritis and hypergastremia, he received the diagnosis of type I gastric NET.

A Japanese, multicentric study retrospectively investigated 82 patients with gastric NET and reported that vascular invasion, muscle-layer invasion, and lymph node metastasis occurred only in 9.8%, 1.6%, and 1.2% of cases, respectively. Furthermore, none of the cases had a distant organ metastasis, and the 5-year survival rate was 100% [7]. The study also demonstrated that a diameter > 10 mm, high Ki-67 score, and muscle layer invasion were independent risk factors of nodal metastasis. Based on these data, the 2019 Japanese guidelines for gastroenteropancreatic neuroendocrine neoplasms recommend a gastrectomy with lymphadenectomy for type I gastric NET only when the tumor fulfills one of the following criteria: diameter > 10 mm, muscle layer invasion or a lymph node metastasis. According to the guidelines, type I gastric NET does not require an invasive intervention until local or regional progression is observed.

On the other hand, two, previous studies raised concerns about the risks of under intervention posed by the administration of endoscopic treatment alone. Approximately 30% of patients with type I gastric NET required further intervention via endoscopic resection or surgery at a rate higher than 50% [8, 9]. Moreover, some cases reportedly developed a lymph node metastasis of gastric NET even after macroscopic curative resection via endoscopic treatment or surgery [10, 11]. These data suggested that type I gastric NET is a slowly growing but potentially malignant tumor requiring curative surgery at any timing.

The European Neuroendocrine Tumor Society (ENETS) guidelines recommend endoscopic surveillance every 12 months in patients with tumor recurrence as the optimal form of treatment, and a surveillance every 24 months for patients with a history of NET with no current tumor recurrence [12]. The National Comprehensive Cancer Network (NCCN) guidelines recommend that patients with small gastric NET (≤ 20 mm) who do not require endoscopic resection or treatment be evaluated using their history and receive a physical examination every 6–12 months. The guidelines also recommend a follow-up endoscopy every 6–12 months for the first 3 years but not thereafter [13]. The present case suggested that routine follow-up may be continued even if no progression is observed during the initial 3 years.

Considering the potential for recurrence after a partial or subtotal gastrectomy, minimally invasive surgery, such as laparoscopic or robotically assisted surgery, is preferable to conventional open surgery, because it can be done with a small incision and causes less adhesion, enabling a second operation to be performed more easily and safely [14]. Moreover, a recent study suggested that robotically assisted surgery has the potential to reduce postoperative complications, especially intra-abdominal complications, to a greater extent than laparoscopic surgery owing to multi-joint articulation and 3D magnification available in the former [15]. This fact gives further support to the use of minimally invasive surgery even in patients with gastric NET requiring surgery.

In conclusion, the present case describes, after a 32-year follow-up period, a patient with multiple gastric NET and a lymph node metastasis underwent a successful robotic-assisted total gastrectomy. Although gastric NET shows a favorable prognosis, long-term follow-up is necessary to detect delayed recurrences and progression.

## Data Availability

The data in this study are available from the corresponding author upon reasonable request.

## Abbreviations

NET: Neuroendrine tumor
ESD: Endoscopic submucosal dissection
CT: Computed tomography
